# Taste Receptors: New Players in Sperm Biology

**DOI:** 10.3390/ijms20040967

**Published:** 2019-02-22

**Authors:** Alice Luddi, Laura Governini, Dorke Wilmskötter, Thomas Gudermann, Ingrid Boekhoff, Paola Piomboni

**Affiliations:** 1 Department of Molecular and Developmental Medicine, Siena University, 53100 Siena, Italy; laura.governini@unisi.it (L.G.); paola.piomboni@unisi.it (P.P.); 2 Walther-Straub-Institute of Pharmacology and Toxicology, Ludwig-Maximilians-University, 80539 Munich, Germany; d.wilmskoetter@web.de (D.W.); Thomas.Gudermann@lrz.uni-muenchen.de (T.G.); ingrid.boekhoff@lrz.uni-muenchen.de (I.B.)

**Keywords:** sperm, taste receptor, spermatogenesis, apoptosis, epididymal sperm maturation, acrosome reaction, knockout mice, reproduction, cAMP, calcium, spontaneous activity of GPCRs, SNP

## Abstract

Taste receptors were first described as sensory receptors located on the tongue, where they are expressed in small clusters of specialized epithelial cells. However, more studies were published in recent years pointing to an expression of these proteins not only in the oral cavity but throughout the body and thus to a physiological role beyond the tongue. The recent observation that taste receptors and components of the coupled taste transduction cascade are also expressed during the different phases of spermatogenesis as well as in mature spermatozoa from mouse to humans and the overlap between the ligand spectrum of taste receptors with compounds in the male and female reproductive organs makes it reasonable to assume that sperm “taste” these different cues in their natural microenvironments. This assumption is assisted by the recent observations of a reproductive phenotype of different mouse lines carrying a targeted deletion of a taste receptor gene as well as the finding of a significant correlation between human male infertility and some polymorphisms in taste receptors genes. In this review, we depict recent findings on the role of taste receptors in male fertility, especially focusing on their possible involvement in mechanisms underlying spermatogenesis and post testicular sperm maturation. We also highlight the impact of genetic deletions of taste receptors, as well as their polymorphisms on male reproduction.

## 1. Taste Receptors and Signal Transduction

The name “Taste receptors” (TAS) derives from their first identification in the oral cavity [1] and their role in the sensation of gustation. Indeed, they were first classified as sensory receptors, whose expression was limited to small clusters of specialized epithelial cells which reside within taste buds located on the tongue [2].

The sensation of taste can be divided into five distinct categories [3]: (i) sweet, for detection of sugars and sweeteners; (ii) salty, for detection of sodium; (iii) umami, for detection of all L-amino acids in rodents [4] but only of *L*-glutamate in humans [5], required by the body for energy balance and building proteins; (iv) sour, which perceives acids in unripe fruit and spoiled foods and (v) bitter, which detects a variety of alkaloid substances, many of which are toxic. However, taste receptors for non-canonical taste stimuli have been described; among them are receptors for kokumi, a stimulus that enhances the basic taste sensations [6] and fatty acid transporters (receptor for fat), involved in oral detection of different fatty acids [7]. Taste sense acts as a guardian and guide for our eating habits: The sensations of bitter and/or sour acts as deterrent ingesting toxic substances and strong acids, while the sensations of sweet, umami and salty lead us to prefer foods containing carbohydrates, amino acids and sodium [8]. Consequently, it is not surprising that the capability to detect and react to chemical stimuli is a trait possessed by the simplest forms of life [9].

Taste transduction signalling involves the interaction of molecules (i.e., tastants) with their specific taste receptors, expressed by cells residing in the taste buds. Taste buds are the transducing endorgans of gustation and each bud comprises 50–100 elongated cells located on the connective papillae of the tongue and scattered throughout the epithelium of the soft palate and larynx. Taste buds are onion-shaped structures. They extend from the basal lamina to the surface of the tongue, where their apical microvilli encounter taste stimuli in the oral cavity, detecting and distinguish between bitter, sweet, sour, salty and umami stimuli.

**Figure 1:**
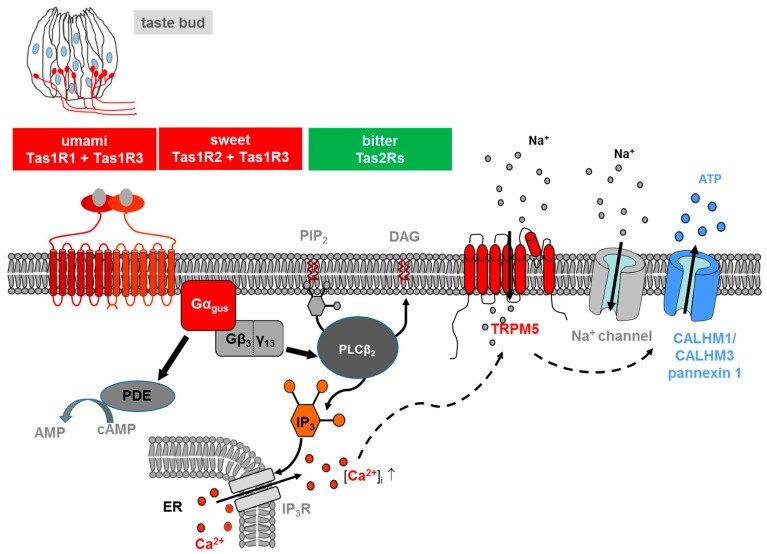
Transduction of *L*-glutamate (umami), sweet and bitter stimuli in taste receptor cells on the tongue. Ligand-induced stimulation of the umami (Tas1r1+Tas1r3), sweet (Tas1r2+Tas1r3) or bitter receptors (Tas2rs) expressed at the apical membrane of type II taste cells within a taste bud (s. drawing in the left) activates in all cases a trimeric G protein composed of α-gustducin (Gα_gus_) and a complex consisting ofGβ_3_ andGγ_13_ (Gβ_3_/γ_13_). The released Gβγ-complex activates phospholipase C isoform β2 (PLCβ2) which then induces production of inositol 1,4,5-trisphosphate (IP_3_) and diacylglycerol (DAG); the second messenger IP_3_, in turn, activates the IP_3_ receptor (IP_3_R), an intracellular ion channel that allows Ca^2+^ release from the intracellular endoplasmic reticulum (ER) store (solid lines). Increase in intracellular Ca^2+^ then activates the transient receptor potential melastatin 5 (TRPM5), a plasma membrane localized sodium-selective channel which leads to depolarization and subsequent activation of voltage-gated sodium channels (Na^+^ channel) (dashed lines). The combined action of elevated Ca^2+^ and membrane depolarization opens the calcium homeostasis modulator (CALHM) channel, composed of CALHM1 and CALHM3 and pannexin1 channels, thus resulting in the release of the neurotransmitter ATP. At the same time, α-gustducin activates a phosphodiesterase (PDE) (solid lines), which catalyses the hydrolysis of the second messenger cyclic-AMP (cAMP) to AMP. For the sake of simplicity, regulatory effects of cAMP are omitted in the model.

Salts and acids utilize apically located ion channels for transduction, while bitter, sweet and umami (*L*-glutamate) utilize G protein-coupled receptors (GPCRs) and a subsequent second-messenger signal transduction process (Figure 1). If compared with other GPCRs, TAS are low affinity receptors, with binding affinities in the micro- to millimolar range, typical for the concentration of most nutrients in foods [10].

Two different families of taste GPCRs have been identified: Type 1 Taste Receptors (Tas1s) and Type 2 Taste Receptors (Tas2s): Tas1s encode the receptor proteins for sweet and umami taste, while Tas2s mediate bitter taste [11,12].

Three different Tas1s have been identified, which are products of the *Tas1s* genes: *TAS1R1*, *TAS1R2* and *TAS1R3* [11,13]. These receptors are activated only if assembled into heterodimers: TAS1R3 heterodimerizes with TAS1R1, thereby forming the umami receptor (TAS1R1 + TAS1R3); assembly of TAS1R3 with TAS1R2 led to the formation of a sweet receptor (TAS1R2 + TAS1R3), activated by carbohydrates, artificial sweeteners and sweet proteins [14,15]. TAS1R3 may also serve as a low-affinity sweet receptor alone, perhaps as a homodimer or homomultimer [16]. The taste 2 receptors, consisting of a large family including about 25 different isoforms in humans and about 35 in rodents, are responsible for the sensation of bitter tastants [12,17,18].

The signalling of both TAS1Rs and TAS2Rs is mediated by the same intracellular transduction pathway in type II taste bud cells [10,19,20] (Figure 1). The binding of the corresponding ligand activates a heterotrimeric G protein, which consists in most cells of the G protein α-gustducin and β_3_/γ_13_, leading to the release of the G β/γ subunits and a subsequent stimulation of phospholipase C isoform β2 (PLCβ2), which, in turn, hydrolyses the membrane lipid phosphatidylinositol 4,5-bisphosphate (PIP_2_) to produce the two second messengers inositol 1,4,5-triphosphate (IP_3_) and diacylglycerol (DAG) [8,10,20]. IP_3_ opens IP_3_ receptor (IP_3_R_3_) type 3 ion channels on the endoplasmic reticulum membrane, thus releasing calcium (Ca^2+^) into the cytosol of the activated receptor cell. As a result, increase in the intracellular Ca^2+^ level activates the cation channel transient receptor potential, melastatin family member 5 (TRPM5) [21,22]. The TRPM5-triggered influx of Na^+^ and activation of voltage dependent sodium channels, subsequently depolarize the cell, leading to a release of the neurotransmitter adenosine triphosphate (ATP) through pannexin 1 and a hexameric channel composed of Calcium homeostasis modulator (CALHM) 1 and CALHM3 [23,24,25]. ATP finally transmits the signal to ionotropic purinergic receptors P2X2 and P2X3 receptors on gustatory afferent fibres [26,27]. Simultaneously released α-gustducin activates phosphodiesterase, thus resulting in a decrease of intracellular levels of the second messenger cyclic adenosine monophosphate (cAMP) [28].

## 2. Expression of Taste Receptors in Different Tissues

Despite their name, the expression of taste receptors is not limited to taste buds in the oropharynx tract [29]. The same is true for the coupling taste transduction cascade which elements are expressed in many chemoresponsive epithelial cells, scattered within both the alimentary tract and the respiratory passageways. Despite the similarities in receptor molecules and the transduction pathway, the emerging picture is that the diverse chemoreceptive cells do not all evoke a sensation of taste but rather serve different functions according to their location [8].

In addition, expression of taste receptors has been reported in many other tissues, both in human and in animals, including the digestive system [16,17,30,31], respiratory system [32,33,34], urinary bladder [35], pancreas [16,36,37], liver [36], brain [38] and testis [5,39,40,41,42,43,44]. In these tissues, taste receptor function seems to be less obvious, and, in most cases has not been clarified yet.

The expression of the Tas1r2 and Tas1r3 subunits in mouse brain, along with the observation that Tas1r2 expression patterns in mouse hypothalamic cells varied according to the glycaemic index of the culture medium, may indicate a direct involvement of these taste receptors in brain glucose homeostasis [38]. From the neonatal stage, human myocytes express the Tas1R3 subunit; anyway, its expression levels are not affected by 24 h of glucose deprivation [45].

There are numerous evidences that taste receptors are involved in the regulation of insulin release, representing an intriguing mechanism alternative to the canonical pathway. Indeed, in addition to the expression of TAS1R2 and TAS1R3 and their coupled G protein α subunit gustducin in the plasma membrane of human β cells, it has been reported that fructose or artificial sweeteners can induce an increase in insulin release more rapidly if compared with the response expected for a metabolic pathway and that this increase is completely abolished by Tas1r3 inhibitors [46,47,48].

As regard to the Tas1r2 and Tas1r3 expression in the bladder urothelium, it has been suggested that they might be involved in bladder contraction induced by artificial sweeteners, such as acesulfame-K or saccharin [35].

A possible involvement of taste receptors in osteogenesis and in bone marrow adipogenesis has been recently proposed [49]: *Tas1r2* knockout mice display a significant decrease in the number of adipocytes in bone marrow together with an increase in bone density. A role for sweet taste receptor in immunity has been hypothesized based on the expression of Tas1r3 in the kidney, in lymphocytes and in thymus [39,50]. Also, bitter receptors are involved in the innate immunity; the rapid response of the epithelial barrier in avoiding infection at the early stage, for example by increasing ciliary beat frequency in order to accelerate mucociliary clearance. It has been reported that human ciliated airway cells express TAS2R4, TAS2R43 and TAS2R46 and ciliary beat frequency is increased by the transduction cascade elicited by TAS2Rs, culminating with a rise in the intracellular levels of Ca^2+^ [32]. Tas2Rs seems to be involved in smooth muscles contraction, too; Tas2r agonists cause the relaxation of pre-contracted airway smooth muscle ex vivo and decrease airway resistance in vivo in mice [51].

From this extensive list it clearly emerges that tasting is only a part of the responsibility of these receptors, which do not mediate “taste” per se as they are not linked to neuronal perceptive pathways. Nonetheless, taste receptors seem to have a chemosensory role in many tissues, which, together with the observation that many medications in clinical use taste bitter and thus are aversive to children [52], opens the way to new therapeutic strategies based on the use of taste receptors as potential therapeutic mediators of drug effects.

## 3. Taste Receptors and Spermatogenesis

### 3.1. Spermatogenesis

**Figure 2:**
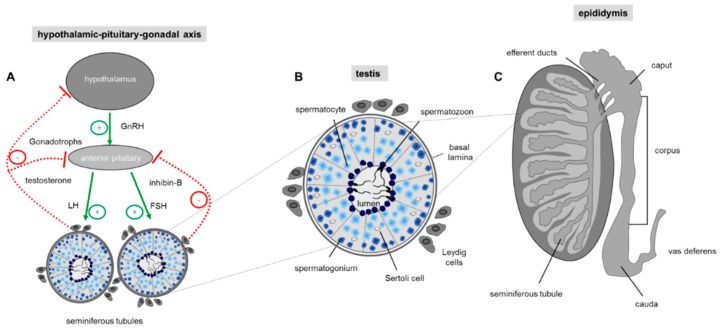
Regulation of sperm production. (**A**) Hormonal control of spermatogenesis in the testis. Spermatogenesis in the testis is under endocrine and paracrine control, which is regulated by the hypothalamus and the pituitary gland also known as hypothalamic-pituitary-gonadal (HPG) axis. The hypothalamus regulates the hormonal activity of the anterior pituitary gland by secreting the tropic gonadotropin-releasing hormone (GnRH). Upon binding of GnRH to the anterior pituitary gland production of luteinizing hormone (LH) and follicle stimulating hormone (FSH) is elevated which upon blood stream transport stimulate testosterone secretion by intestinal Leydig cells and activation of Sertoli cells by FSH. Sertoli cells as cellular part of the tubular unit provide the optimal environment for the developing germ cells. A negative feedback of GnRH production in the hypothalamic neurons and LH/FSH secretion by the pituitary gland is exerted by high testosterone levels in the blood and secretion of the proteohormone inhibin-B by Sertoli cells. Arrow: positive (green) and negative (red) feedback. (**B**) Schematic drawing of a single seminiferous tubule with different stages of developing germ cells during spermatogenesis. The cross section shows that germ cells of a distinct developmental stage are organized in concentric layers within the tubule: In the most basal cell layer of the tubular unit, the immature spermatogonial stem cells are located, followed by spermatocytes, round spermatids and finally the most mature elongated spermatids which are concentrated in the lumen of the seminiferous tubule. The regulation of spermatogenesis is mainly mediated by surrounding interstitial Leydig cells which produce testosterone. The Sertoli cells within the seminiferous tubules have a nurturing role for the developing germ cell and transduce the action of FSH to the closely associated germ cells. (**C**) Schematic drawing showing a sagittal section through a whole testis and the overlying epididymis. The testis contains the tightly packed seminiferous tubules where spermatogenesis takes place. The elongated duct presenting the epididymis at the posterior margin of the testis is subdivided into three discrete segments (caput, corpus, cauda), where the luminal fluid of each region is characterized by a unique composition of different constituents, essential for post-testicular sperm maturation.

Spermatogenesis is a complex and precisely controlled cellular transformation process that ensures the production of millions of sperm daily [53]. This massive sperm production takes place in the tightly packed seminiferous tubules of the two testes where each tubular unit contains distinct concentric layers of germ cells of different stages of maturation (Figure 2): Diploid spermatogonia, the stem cells of the testis, are localized in the basal cell layer of the seminiferous tubules. Upon mitotic divisions that provide the necessary cell number essential for a high sperm output, developing spermatocytes move to the more luminal part of the seminiferous tubule where they undergo meiosis resulting in the generation of haploid spermatids. The round spermatids subsequently run through a cellular transformation process called spermiogenesis in which they differentiate into spermatozoa finally localized into the luminal region of the tubular unit [54].

Continuous sperm production in adult males depends on endocrine and testicular paracrine/autocrine factors which together coordinate proliferation and germ cell differentiation [55,56]. The endocrine stimulation of spermatogenesis involves the two gonadotropins follicle stimulating hormone (FSH) and luteinizing hormone (LH). Their secretion by the anterior pituitary gland is controlled through the hypothalamic-pituitary portal system with gonadotropin-releasing hormone (GnRH) secreted by the hypothalamus [55] (Figure 2A). Subsequent action of the two glycoproteins LH and FSH requires cell to cell communication within the testis which is predominantly mediated by the two somatic cell types within the testis, the Leydig and Sertoli cells. Leydig cells, that reside between the seminiferous tubules of the testis, produce testosterone upon LH stimulation [57]. Sertoli cells, which form cytoplasmic bridges with the developing germ cells within the seminiferous tubules, are the ‘nurse’ cell of the testes [58] and play a more comprehensive role: Sertoli cells create the adequate ionic environment for germ cell development, have a nurturing role for differentiating sperm, phagocytose residual bodies after spermiogenesis and assist in the final migration of mature spermatozoa into the lumen of the seminiferous tubule [59]. In addition, since the germ cells do not possess receptors for FSH and testosterone, Sertoli cells represent the major cellular targets for hormonal signalling so that the effect of hormones on germ cell development is indirect [60]. The hypothalamic-pituitary-gonadal (HPG) axis is a self-regulating system with two negative feedback loops (Figure 2A): on one hand high testosterone concentrations in the peripheral blood provides a negative feedback route to suppress hypothalamic discharge of GnRH and consequently LH release from the anterior pituitary [61]. The second loop is the release of inhibin-B by Sertoli cells. Inhibin-B has a negative feedback effect on the pituitary gland, thereby suppressing FSH secretion [62] (Figure 2A).

### 3.2. Apoptosis

However, success of germ cell proliferation and differentiation is also ensured by a dynamic balance between germ cell development and a carefully controlled process of programmed cell death, thereby ensuring a selective elimination of an overrun of produced germ cells and in addition a deletion of abnormal and defective sperm [63]. Removal of an excess of germ cells taking place during spermatogenesis in the testis and ensuring an optimal ratio of supporting Sertoli cells to germ cells during at all stages of development leads to a degeneration of about 75% of spermatogonia before reaching maturity [64,65]. In maturated and ejaculated sperm where apoptosis also occurs [66] the process of programmed cell death is responsible to eliminate damaged cells [64]. Any imbalance in the apoptotic process has dramatic implications for male infertility: whereas a decrease in the selective elimination of defective developing and mature sperm causes poor sperm quality, an increase in apoptosis could potentially lead to a reduced sperm count and thus sub-fertility [67,68]. Thus, identification of “death triggering signals” [69] as well as corresponding receptor proteins that elicit activation of the apoptotic machinery is of critical importance for the fertilization potential of males. Although not fully understood [64], onset of apoptosis in germ cells can not only be induced by the lack of hormones, like gonadotropins and testosterone [56] but also by a broad range of non-hormonal and also non-physiological stimuli, such as heat stress, industrial and therapeutic agents as well as a variety of naturally occurring toxicants [70,71]. In this context one has to consider that receptors belonging to the taste 2 family are specialized to detect bitter compounds including extremely toxic alkaloids [72,73]. Since genes for all 35 bitter receptors have been identified in mouse testis [74] bitter receptors might present promising candidates to detect testicular toxicants. Moreover, genetic deletion of the *Tas1r1* receptor, the dimerization partner of the Tas1r3 which in taste buds on the tongue forms the functional receptor for *L*-glutamate (umami), leads to a significant increase in the number of apoptotic cells during spermatogenesis [42], an observation that already indicates that taste receptors indeed play a functional role for controlling apoptosis in the male reproductive tissue.

## 4. Taste Receptors in Epididymal Maturation and Sperm Function

### 4.1. Epididymal Sperm Maturation

After spermatids have completed the last developmental stage of spermatogenesis in the testis which in humans takes 65 days [75], the morphologically complete but still immature germ cells travel to the epididymis. The epididymis represents an elongated but structurally segmented duct connecting the testis and the vas deferens (Figure 2C) where the most distal caudal region is responsible to store fully mature spermatozoa until ejaculation occurs [76]. Importantly, acquisition of motility of sperm and their ability to penetrate and fertilize the female gamete only occurs during their entire transit through the three anatomical segments of the epididymis (caput, corpus and cauda part of the epididymis) (Figure 2C), a process called epididymal sperm maturation [77,78].

Remarkably, from the proximal to the distal end of the long epididymal tubule luminal fluid microenvironment surrounding the spermatozoa progressively changes [79]: this includes a decline in sodium and a rise in potassium ion concentrations, a shift in pH from the acidic range at the caput to the alkaline at the cauda part of the epididymis [80] and a decrease in bicarbonate (HCO_3_^−^) [81]. Moreover, a progressive decline in the free Ca^2+^ concentrations in the distal part of the epididymal duct has been registered where luminal Ca^2+^ is absorbed by the Ca^2+^ selective channel transient receptor potential vanilloid (TRPV6) [82].

Interestingly, during this transit through the epididymis, sperm are exposed to various molecules of the seminal plasma that, by adhering to the sperm membrane, prevent untimed acrosome reaction until they are removed in the female genital tract [83,84]. To this regard, particular attention deserves the role of cholesterol, a very important component of higher eukaryotic cell membranes, and, especially for sperm. In male gametes cholesterol is fundamental in the membrane dynamics and functionality, where it is known to regulate GPCRs through direct or indirect interactions [85,86]. Most of the GPCRs present a cholesterol recognition/interaction amino acid consensus (CRAC) motif as a molecular determinant required for interaction with cholesterol [87]. Even if several aspects have to be clarified, the presence of a CRAC motif in Tas2Rs argues for cholesterol dependent signalling functions of Tas2Rs, through stabilization of these receptors in the membrane and/or modulation of their function [87,88]. This evidence supports a possible involvement of taste receptors in the process of epididymal sperm maturation.

### 4.2. Sperm Function: Capacitation, Motility, Chemotaxis, Acrosome Reaction

**Figure 3:**
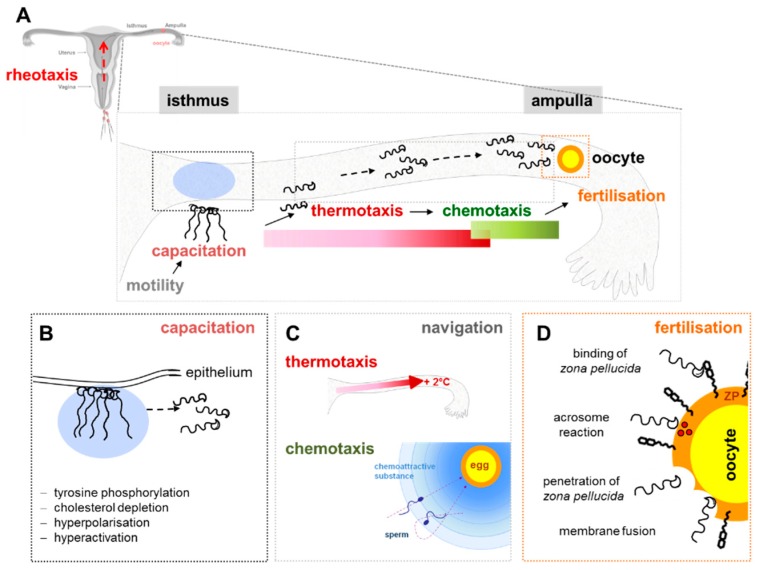
Schematic drawing showing the most critical steps during the sperm’s transit through the female genital tract before fertilizing the egg. The gamete interactions are a critical step on reproduction. Mammalian fertilization comprises: (i) sperm migration through the female reproductive tract (rheotaxis, thermotaxis and chemotaxis), (ii) biochemical and morphological changes to sperm (capacitation) and (iii) sperm-egg interaction in the oviduct (fertilization) (**A**). In the female reproductive tract, specifically in the isthmus of the uterus, the mammalian sperm must undergo a series of important modifications, such as tyrosine phosphorylation, cholesterol depletion, hyperpolarisation and finally hyperactivation. These complex priming processes, by which sperm become competent to fertilize an egg, are all together termed “capacitation” (**B**). Chemotaxis permit sperm to move into the ampulla and locate the egg, *organized in a cell complex* (**C**). The ovulated oocyte is covered by a multicellular cumulus oophorous. The fertilization takes place after specific steps: (*i*) binding of *zona pellucida*, (*ii*) acrosome reaction, (*iii*) penetration of zona pellucida and (*iv*) final membrane fusion (**D**).

In recent years it has become obvious that ejaculated sperm cannot reach the oocyte just by chance. In fact, even if in humans hundreds of millions of sperm enter the female tract upon ejaculation, only a small fraction (about 10^4^) enters the oviduct [89]. This drastic depletion, due in part to the many obstacles encountered by sperm during its journey but also to its small size relative to the female genital tract that must be traversed, makes the random encounter of sperm and oocyte unlikely to occur. To explain this phenomenon other than by “luck,” three different mechanisms of sperm guidance have been suggested: (i) thermotaxis or swimming up a temperature gradient, (ii) rheotaxis, that is swimming against a fluid flow and (iii) chemotaxis (Figure 3) [89,90,91,92,93]

Chemotaxis, the most intriguing and studied guidance mechanism, is defined as the ability of sperm to swim towards a gradient of chemical factors, also named chemoattractants, whose chemical nature, as well as their cognate receptors, are still not fully understood. Chemotaxis can be affected by two physiological processes that sperm undergo in the female genital tract, namely capacitation and hyperactivation [94,95,96]. Capacitation is a maturation process making sperm able to penetrate cumulus cells, to bind to the sperm receptor of the zona pellucida and to undergo acrosome reaction [97], even if some authors suggest that acrosome reaction in sperm able to fertilize the oocyte begins during cumulus cell penetration [98]. Membrane remodelling, a prerequisite for acrosome reaction, is induced by cholesterol efflux, that alters lipid raft stability and distribution, favours specific protein-protein interactions by concentrating certain proteins in specific microdomains while excluding others. The physiological cholesterol efflux seems to play a regulatory role on taste receptors through the CRAC motif, influencing TasRs signalling efficacy, as reported in human airway cells [99].

In addition, capacitation process is mandatory for the acquisition of shortrange chemotactic responsiveness [95,100]. During capacitation sperm undergo a change in the motility pattern called hyperactivation, characterized by vigorous, whip-like flagellar movement, that allow sperm release from the oviductal reservoir and the penetration of the layers surrounding the oocyte [101,102,103,104]. Twenty-five years after the demonstration that human follicular fluid contains substances causing sperm chemotaxis in vitro [105], the role of progesterone as chemoattractant, controlling sperm navigation and fertilization has been demonstrated [106,107]. Progesterone activates CatSper ion channel causing a Ca^2+^ influx [92,102,103]. Anyway, many aspects of this complex mechanism possibly involving taste receptors still need to be clarified.

The analysis of the signal transduction cascade elicited during chemotactic activation, along with the chemical nature of chemoattractantsand the reported expression of taste receptors in sperm, leads to hypothesize about an involvement of these highly specialized receptors in this process. In fact, analogously to what previously stated for taste receptors, it has been demonstrated that signal transduction in chemotaxis involves modulation of adenylate cyclase or phospholipase C by appropriate GPCRs [108]. Increasing amounts of cAMP induce sperm oxygen consumption and motility. At the same time, a rise in concentration IP_3_ causes the release of Ca^2+^ from the acrosome and from stores in the midpiece, that, in turn, modulates sperm motility by inducing flagellar beat asymmetry, probably mediating the mammalian chemotactic response [109,110]. By means of this complex transduction pathway, sperm seem to use different chemical prompts to spot the oocyte. Interestingly, signalling in sensory neurons and sperm involves analogous substances, with the signalling occurring in the “cilium,” that in sperm is indicated as flagellum. Like neurons, sperm express on their equatorial segment membrane GABA A receptor [111,112], through which they interact with GABA, shown to be present in rat oviduct and human seminal plasma, which causes hyperactivated motility [112,113].

Moreover, insights from the airway showing a strong expression of taste receptors on motile cilia confirms that these can also play a role in cell chemosensation [114]. As a consequence, the flagellum seems to be fundamental in guiding sperm through the microenvironment rich in chemical and physical stimuli that they encounter.

Sperm-activating odorous substances have been identified in human follicular fluid [115] and, analogously to taste receptors, also odorant receptors (OR) have been identified in tail and midpiece regions of ejaculated sperm [116,117]. The human testicular odorant receptor hOR17-4 induces in sperm a significant increase in calcium intracellular level, when stimulated by different ligands, suggesting a key role for OR in guiding sperm toward oocyte [118].

Unpublished results from our lab disclose an expression of members of the Tas1R as well as Tas2R receptor family in ejaculated human sperm. Importantly, after an in vitro capacitation, a change in the subcellular expression pattern was detectable in immunocytochemistry, both at the head and tail sperm level. This reorganization may be related to a preceding activation of taste receptor proteins and a subsequent membrane remodelling, a process well known to be associated with sperm capacitation. In addition, we disclose that in vitro capacitation is associated with the presence of different isoforms of GCPRs, as demonstrated by western blot analysis.

Data from the literature demonstrate that the metabotropic glutamate receptor taste-mGluR5, belonging to the GPCR family, is expressed not only in the membranes of the taste cells of the taste buds but also in testis and in mature sperm, where it was localised in the mid-piece and in the tail [119] together with beta β-arrestin, a protein involved in the homologous desensitisation and internalisation of GCPRs [120]. Interestingly, it has been reported that the glutamate concentration increased in follicular fluid when compared with plasma [121] making this compound eligible as a chemoattractant molecule. These data, together with the previously described evidences from neurons and airway, one may hypothesize a role for these receptors in guiding sperm during their journey toward the oocyte.

The ability of sperm to fertilize the egg is acquired during capacitation and this includes the capability to undergo the acrosome reaction, which is a regulated exocytotic process leading to an extensive fusion between the outer acrosomal and the plasma membranes of sperm, enabling the male gamete to penetrate the zona pellucida and fuse with the oocyte. Capacitation is a HCO_3_^−^ and Ca^2+^-dependent process. Ca^2+^ triggering is fully mediated by the increase of cAMP [122]. The second messenger cAMP is able to activate the protein kinase A (PKA) which in turn phosphorylates certain proteins on tyrosine; other cAMP-binding proteins, such as Rap guanine-nucleotide-exchange factor (Epac) and the cyclic-nucleotide-gated ion channels, are also responsive to cAMP [123]. It has been demonstrated the presence of EPAC in human sperm, crucial in the pathway of exocytosis downstream of Ca^2+^ [123]. The balance between PDEs and adenylyl cyclases (ACs) controls cAMP levels, by degrading or producing the molecule, respectively. A soluble AC (sAC) now named atypical adenylyl cyclase (SACY) and a transmembrane AC (tmAC) have been detected in mammalian sperm: HCO_3_^−^ as well as Ca^2+^ stimulate the SACY thereby enhancing cAMP concentrations [124]. The function of specific tmACs in sperm is not fully understood. It has been suggested that tmACs may have a role in the acrosome reaction due to the presence of different G-protein α subunits in the sperm acrosome, since tmACs are regulated by G proteins [41,125]. Moreover, G proteins α-subtypes gustducin and transducin have been detected in the acrosome of mammalian sperm, including human [126,127] (unpublished data from our group); in addition some G protein α-subtypes are expected to be able to activate a phosphodiesterase, decreasing the intracellular level of cAMP [10], as reported for the visual system. Therefore, some authors [42,128] suggested that both α-gustducin and α-transducin may modulate cAMP levels in sperm, thus contributing to regulate the acrosome reaction process and avoiding a precocious acrosome lost during the journey on the female genital tract, where umami, bitter and sugar stimuli may active taste transduction signals. This last hypothesis was affirmed by Meyer and colleagues [42]: using a Tas1r1/mCherry reporter mouse line; they demonstrated that Tas1r1 null-mutant sperm have an increased degree of spontaneous acrosome reaction as well as higher intracellular Ca^2+^ and cAMP levels.

## 5. Genetic Deletion of Taste Receptors in Mouse and its Impact on Male Reproduction

During spermatogenesis and sperm’s journey through the epididymis and the female genital tract sperm are exposed to a vast variety of chemical compounds in the surrounding milieu, such as hormones, changes in pH, amino acids, proteins, sugar gradients but also potential toxicants. The recent observations that taste receptors and elements of the coupling signal cascade are expressed in developing germ cells but also in mature spermatozoa ranging from mouse to humans (Section 2) as well as the striking overlap of the ligand spectrum of taste receptors with compounds in the natural surroundings of germ cells now makes it conceivable that sperm recognize these different cues in their natural microenvironments.

**Figure 4:**
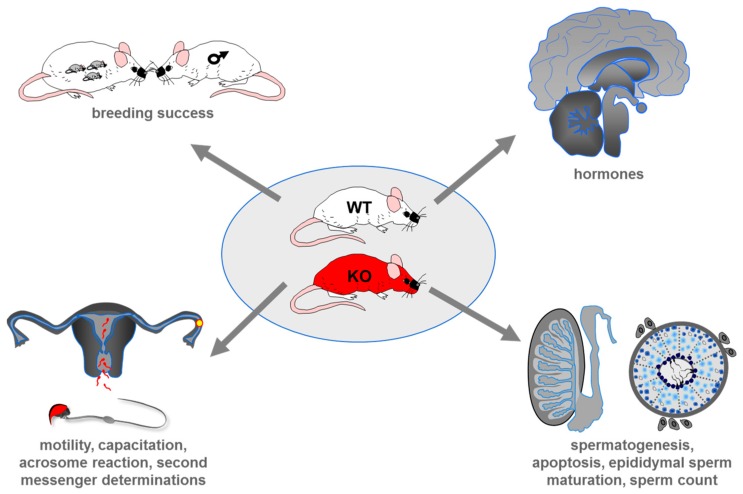
Experimental strategy to determine the impact of taste receptors for male reproduction. To gain a complete picture about a possible role of taste receptors for regulating male reproduction standard reproductive parameters (e.g., litter size, time to litter, sex ratio of pubs) were determined for breeding pairs of wild-type [WT] and taste receptor deficient animals [KO]. To evaluate whether genetic deletion of taste receptors affect spermatogenesis results of breeding experiments were supplemented by histopathological examinations of reproductive organs and isolated epididymal sperm cells; furthermore, reproductive-related hormones such as testosterone, LH, FSH and GnRH were quantified. To evaluate whether a loss of taste receptors modifies physiological sperm function, CASA (computer-assisted motility analysis) -based motility analyses were combined with experiments assessing the ability of sperm to respond to capacitation and acrosomal exocytosis stimuli.

To clarify whether taste receptor proteins indeed represent molecular “sensors” in the male reproductive system the powerful gene targeting strategy to produce taste receptor deficient mouse lines has been used. Such mouse lines allow to directly evaluate the physiological impact of taste receptors for successful reproduction by combining systematic breeding experiments with morphometric analyses of the testis, epididymis and male germ cells, quantitative determinations of reproductive-related hormones and second messengers (cAMP, Ca^2+^) as well as functional sperm tests (Figure 4).

To date, two studies have been published describing the reproductive phenotype of mouse lines carrying a genetic deletion of taste receptor proteins: A reporter mouse strain in which the open reading frame of the umami receptor gene, the *Tas1r1*, was replaced by a fluorescent protein (mcherry) [42]. Such a genetic labelling of a target null mouse model additionally enables visualizing ectopic expression at a single cell resolution [43]. Moreover, a double knockout mouse line carrying a simultaneous genetic deletion of the dimerization partner of the umami as well as sweet taste receptor protein, the Tas1r3, together with the G protein α-subunit gustducin was engineered [129].

Importantly, produced Tas1r1/gustducin double knockout mouse strain was also used to generate a humanized chimeric (hm) Tas1r3 form, thus allowing a specific inhibition of the taste receptor protein by the lipid-lowering agent clofibrate, an antagonist for the human but not the mouse Tas1r3 [130,131,132]. The recently published manuscript of the sweet taste receptor Tas1r2 knock-in mouse strain was only used imaging extragustatory sweet receptor expression (Section 2); a reproductive phenotype of this mouse line has not been described yet [50]. The same is true for an engineered Tas2r5 bitter receptor knockout mouse line where testicular expression was optically displayed by the death of Tas2r5 expressing cells induced by the diphtheria toxin A [5].

Comparing the results of these two taste receptor knockout mouse lines, the striking observation was made that taste receptors apparently play a key functional role in different steps of the sequential process of fertilization ranging from the production of spermatozoa, the induction of apoptosis in the testis to epididymal sperm maturation; moreover, functional implications have been collected indicating that taste receptors are also important to increase the number of highly fertilization-competent sperm cells within the female genital tract.

To first have a look on the reproductive phenotype of the umami receptor deficient mouse strain it was observed that animals lacking the Tas1r1 receptor exhibit no apparent abnormalities and display no severe reproductive phenotype concerning litter size or the number and morphology of epididymal sperm [42]. However, histopathological evaluations of the testis of Tas1r1 gene-knockout males revealed some spermatogenic abnormalities: relative to wild-type males, in mutant testis immature spermatocytes were also visible within the luminal region of the seminiferous tubules instead of being restricted to the more basal cell layer of the testicular unit (Figure 2C). Moreover, an increase in the number of multinucleated giant cells, a histological change characteristic for cells undergoing necrosis or apoptosis was visible in single mutant seminiferous tubules [42,133,134].

An even more pronounced spermatogenic but also post-gonadal impairment was detected for males carrying the concurrent genetic deletion of the Tas1r3 together with α-gustducin. Although Tas1r3/gustducin null males appeared healthy with no changes in the size of their reproductive organs, Mosinger and his colleagues made the unexpected observation that males but not females were unable to produce pubs. Comprehensive histological analyses then uncovered exfoliated germinal epithelium in the luminal part of the testicular tubules; in addition an increase in the number of giant cells with condensed chromatin was detected in the spermatid cell layer of the seminiferous tubules. A comparable severe pathology was visible for the epididymis of Tas1r3/gustducin null males which was found to store mainly immature germ cells and cellular debris within their luminal region. Moreover, a pronounced oligospermia with more than 75% of the remaining sperm being immotile was registered; residual Tasr1/gustducin double knockout sperm were additionally characterized by multiple anatomical abnormalities, such as detached or amorphous heads, tails flipped over heads and multiple kinks and loops in the sperm tails [129]. Importantly, male mice expressing the humanized hmTas1r3 chimera were found to be fertile; however, after a 3 weeks diet supplementing the food with the drug clofibrate the males became sterile due to abnormalities in spermatogenesis, accompanied by malformed and fewer sperm as observed for Tas1r3/gustducin deficient males whereas switching to normal diet males regain fertility within 2 weeks [129].

Due to the pronounced malformation of mature Tas1r3/gustducin null sperm it was redundant to evaluate semen quality, usually captured by quantifying hyperactivated motility, stimulus-induced capacitation and acrosomal secretion (Figure 4). However, for normal-shaped Tas1r1 deficient sperm a significant increase in an “accidental” spontaneous loss of the acrosomal vesicle was registered compared to wild-type littermates whereas sperm motility was not affected [42]. Importantly, taste cells on the tongue of gustducin null mice have previously been found to exhibit elevated stimulus-independent basal concentrations of the second messenger cAMP which seems to be due to a constant lack of α-gustducin triggered activation of a PDE [28]. A comparable basal increase in cAMP was also detected for Tas1r1 deficient epididymal sperm cells, which was also caused by a lack of PDE-dependent cAMP degradation [42].

Remarkably, cAMP but also calcium, likewise elevated in Tas1r1 null sperm [42], are responsible to prepare sperm to reach and bind to the eggs coat, the zona pellucida and to finally cross the egg‘s protective glycoprotein matrix by releasing hydrolysing enzymes during the process of acrosome reaction (for review see [135,136,137]. Since early and unintended acrosomal exocytosis renders sperm infertile [122] it could be possible that Tas1r1 and its coupled downstream signalling effectors in mammalian sperm are constantly activated by the multitude of environmental cues during the sperm’s transit through the female genital tract. This would go along with a persistent suppression of cAMP and calcium triggered events and hence a depression of a loss of the one and only acrosomal vesicle [138]. At the fallopian tube where the mature egg is waiting for fertilization, other cAMP dependent maturation processes, such as bicarbonate triggered activation of sAC (for review see [122]) then may override Tas1r1 receptor signalling, hence ensuring successful fertilization. Although the umami receptor dimer of mouse was found to be a more broadly tuned L-amino-acid receptor activated by most L-amino acids [4], monosodium glutamate, one of the natural ligands of the Tas1r1/Tas1r3 on the tongue, was not capable to elicit calcium signals in capacitated spermatozoa [42], thus suggesting that the Tas1r1 in germ cells probably detects different stimuli than its homologue in taste buds on the tongue. However, a constitutive activation of taste receptors has also been described [139,140]. In this context it is important to note that clofibric acid, the active metabolite of clofibrate [141] acts as an inverse agonist on the human TAS1R3 receptor [129,130]. Due to the pronounced reproductive phenotype of clofibrate treated males expressing the humanized hmTas1r3 on a gustducin null background [129], one might suggest that taste receptors show a high frequency of spontaneous activation [142] and that such a constitutive activity of taste receptors in the male reproductive system is sufficient to fulfil their physiological function.

However, in this context it is additionally important to mention that loss of Tas1r1 also led to a significantly higher level of apoptotic events during spermatogenesis [42]. Although the pathology of the Tas1r3/gustducin deficient double knockout males was found to be very similar but only much more pronounced than the one of the umami receptor null males, an increase in the number of apoptotic cells was not registered for testicular tissue of males expressing the humanized hmTas1r3 on the null gustducin background and clofibrate diet [129]. However, one has to keep in mind that clofibrate treatment only lasted 1 month whereas genetic deletion of Tas1r1 occurred entirely from birth; moreover, males carrying a genetic deletion of Tas1r3 together with gustducin have not yet been examined for apoptosis [129]. However, males carrying a genetic deletion of Tas1r3/gustducin as well as humanized hmTas1r3/gustducin knockout males on clofibrate both show a reduction in the level of various genes known to be regulated by the transcriptional activator cAMP responsive element modulator (CREM). Remarkably, knockout males for CREM are characterized by the completely absence of late spermatids and a significant increase in apoptotic cells which thus also led to sterility of the animals [143]. Due to the similarity of the histological phenotype of Tas1r3/gustducin and CREM null males as well as the observation that CREM is highly expressed in postmeiotic cells of the testis [144,145] one might suggest that CREM, via a taste receptor controlled cAMP pathway, is responsible in ensuring exactness of germ cell development. Although it has to be scrutinized whether cAMP levels are indeed increased in testicular tissue of taste receptor deficient males such a cAMP dependent mechanism may also shed new light on the extragustatory function of mammalian taste receptors. Moreover, since bitter taste receptor share the same signal transduction cascade than Tas1Rs (Figure 1) it will be exiting to prove whether deletion of bitter receptors, also expressed in the male reproductive system (see above), results in the same reproductive phenotype as the one observed for Tas1r knockouts.

## 6. Polymorphisms in Taste Receptor Genesand Male Infertility

Taste receptors genes are highly polymorphic and some SNPs have been correlated with an altered gene expression. Among others, three SNPs in the *TAS2R38* gene, responsible of the ability to taste the bitter compounds phenylthiocarbamide (PTC) and 6-n-propylthiouracil (PROP), can completely alter the individual ability of tasting the substance [146]. In fact, these three SNPs (rs714598, rs1726866 and rs10246939) determine three amino acid substitutions at positions 49 (Proline- Alanine), 262 (Alanine Valine) and 296 (Valine-Isoleucine) that define the taster PAV (Proline, Alanine, Valine) and “non-taster” (Alanine, Valine, Isoleucine) haplotypes. “taster” and “non-taster” individuals show a different intake of several vegetables [147] and sweet food [148]. The missense variant Arg299Cys of the *TAS2R19* gene (rs10772420) is instead associated with a differential taste of quinine and grapefruit juice [149].

Therefore, SNPs in taste receptors influence several human traits and complex diseases, such as drinking behaviour, nicotine dependence, food and beverage choices, body mass index, susceptibility to cancer and human aging [147,148,150,151,152,153].

Recently, the possible link between SNPs in taste receptors and male infertility has been investigated. Four GWAS [154,155,156,157] and a big association study with 172 polymorphisms [158] have been published. In these studies, thirty-nine marginally (*p*-value < 0.05) significant associations were identified in the Caucasians population and six significant (nominal *p*-value < 5 × 10^−8^) in the Chinese population. Interestingly, Aston and colleagues found that the *TAS2R38*-rs10246939 SNP was associated with risk of being azoospermic but this datum was not replicated in two following studies [159,160] and it one must keep in mind that this gene does not seem to be expressed in the human testis and in spermatozoa. Alongside the GWAS many association studies have been carried out and, to date, in the literature, approximately 269 risk variants have been proposed [161]. However, despite the identification of an increasing number of environmental and genetic risk factors the aetiology remains unknown in almost half of the cases [162].

In the last years, SNPs in taste receptors seem to have a significant role in male infertility. Some authors [160] have found some SNPs in taste and odorant receptor genes, which may play a role in spermatogenesis alterations observed in Persian idiopathic infertile male. The associations reported in this study are promising but much larger studies will be necessary to confidently validate these SNPs and identify novel SNPs associated with male infertility. Recently, a study from our research group demonstrated that the homozygous carriers of the (G) allele of the TAS2R14-rs3741843 polymorphism showed a decreased sperm motility compared to heterozygotes and (A) homozygotes and that the homozygous carriers of the (T) allele of the TAS2R3-rs11763979 SNP showed fewer normal acrosome compared with the heterozygous and the homozygous carriers of the (G) allele [163]. In addition, by in silico analyses, we demonstrated a functional effect of the two SNPs: TAS2R14-rs3741843 in regulating TAS2R43 expression. Since this latter is known to be expressed in the human airway epithelia where it is involved in the regulation of ciliary movements to eliminate toxic substances [32], we suggested that it could participate in sperm motility. The proven infertility due to abnormally structured flagella in man suffering from some genetic diseases characterized by a ciliary dysfunction seems to support this hypothesis [164,165].

Moreover, in this study [163] we highlighted that the *WEE2* antisense RNA one gene (*WEE2-AS1*) expression is increased by the (T) allele of TAS2R3-rs11763979. Since *WEE2* gene is expressed in the testes, where presumably it has the role of down regulating meiotic cell division, it is conceivable to assume that an increased expression of *WEE2-AS1* may inhibit *WEE2*, which in turn can alter the natural timing of sperm maturation increasing the number of abnormal sperm cells. To our knowledge, this is the largest study so far reported in the Caucasian population focused on male infertility and genetic variability in taste receptor genes.

## 7. Conclusions

Although the precise molecular mechanism of taste receptor action for reproduction is only poorly understood fertility impairment of mice carrying a genetic deletion of Tas1Rs imply that taste receptor are functionally operative in controlling successful sperm production as well as increasing the chance of a single sperm of the roughly 100 to 300 million in an ejaculate to fuse with a mature egg. Moreover, a role of the genetic variability of taste receptors in human male infertility has been demonstrated, even if these results are not validated by in vitro or in vivo experiments.

Therefore, a better understanding of the precise role of taste receptors in human male fertility is needed which especially concerns the natural ligands for taste receptors in the male and female reproductive organs as well as our knowledge about a spontaneous activity of taste receptor proteins. This information in the future may not only uncover new ways to address idiopathic male and female sterility but may also pave the way to develop novel therapeutic strategies or new methods of contraception.

## Abbreviations

ATP	Adenosine triphosphate
cAMP	Cyclic adenosine monophosphate
HCO_3_-^-^	Bicarbonate
Ca^2+^	Calcium
CALHM	Calcium homeostasis modulator
CASA	Computer-assisted motility analysis
CatSper	Cation channel of sperm
CRAC	Cholesterol recognition/interaction amino acid consensus
CREM	cAMP responsive element modulator
DAG	Diacylglycerol
ER	Endoplasmic reticulum
FSH	Follicle stimulating hormone
GABA	Gamma-aminobutyric acid
GnRH	Gonadotropin-releasing hormone
GPCRs	G-protein-coupled receptors
HPG	Hypothalamic-pituitary-gonadal axis
IP_3_	Inositol 1,4,5-triphosphate
IP_3_R	Inositol 1,4,5-trisphosphate receptor
KO	Knock-out
LH	Leutinizing hormone
Na^+^	Sodium
OR	Odorant receptor
PDE	Phosphodiesterase
PIP_2_	Phosphatidylinositol 4,5-bisphosphate
PLCβ2	Phospholipase C β2
P2X2/3	Purinergic receptor
PROP	6-n-propylthiouracil
PTC	Phenylthiocarbamide
SNP	Single-nucleotide polymorphism
TAS	Taste Receptor
TAS1s	Type 1 taste receptors
TAS2s	Type 2 taste receptors
TRPM5	Transient receptor potential, melastatin family member 5
TRPV6	Transient receptor potential vanilloid 6
WEE2-AS1	WEE2 antisense RNA one gene
WT	Wild-type
